# Editorial: Immunogenic Cell Death in Cancer: From Benchside Research to Bedside Reality

**DOI:** 10.3389/fimmu.2016.00110

**Published:** 2016-03-29

**Authors:** Abhishek D. Garg, Patrizia Agostinis

**Affiliations:** ^1^Cell Death Research and Therapy (CDRT) Laboratory, Department of Cellular Molecular Medicine, KU Leuven University of Leuven, Leuven, Belgium

**Keywords:** immunogenicity, cancer immunology, cancer immunotherapy, anticancer vaccines, damage-associated molecular patterns, danger signals, danger signalling, immunosurveillance

**The Editorial on the Research Topic**

**Immunogenic Cell Death in Cancer: From Benchside Research to Bedside Reality**

Immunogenic cell death (ICD) has emerged as a cornerstone of therapy-induced antitumor immunity (1–3). ICD is distinguished by spatiotemporally defined emission of danger signals or damage-associated molecular patterns (DAMPs) that elevate the immunogenic potential of dying cells [Garg et al.; (4)]. The important role played by DAMPs in immunity, tissue remodeling, and inflammation is discussed in details by Venereau et al. (Marco E. Bianchi lab).

Most potent ICD inducers, characterized so far, elicit danger signaling through oxidative-endoplasmic reticulum stress (5). Several ICD inducers have been characterized, e.g., some chemotherapies, some physicochemical therapies (e.g., radiotherapy or photodynamic therapy/PDT), and oncolytic viruses (2, 6). Here, radiotherapy is among the first recognized immunogenic therapies [on account of “abscopal-effect” (7)]. The immunogenic potential of radiotherapy and possibilities for its combination with immune checkpoint blockers is discussed by Derer et al. (Udo S. Gaipl lab). It is noteworthy that ICD can also be achieved by various “smart” combinatorial strategies – an important point for clinically applied non-ICD inducers, discussed in details by Bezu et al. (Guido Kroemer lab).

Several lines of experimental evidence have established the validity of ICD. However, the overreliance on usage of prophylactic vaccination in transplantable (heterotopic) tumor models has attracted some criticism (8). While these criticisms are valid, the field is already moving toward tumors produced orthotopically (curative/therapeutic) or in genetically engineered mouse models (GEMM) (at least for few ICD inducers, e.g., hypericin-PDT, Newcastle disease virotherapy and anthracyclines) (9–12). Moreover, the clinical existence of ICD has been proven through retrospective analysis involving cancer patient’s survival/therapy-responsiveness data (13–17). These observations have encouraged the increased usage of ICD-associated DAMPs as predictive/prognostic biomarkers – a point discussed in detail by Fucikova et al. (Radek Spisek lab). The promising results generated by systemically administered ICD inducers have also paved way for application of ICD-based dendritic cell (DC) vaccines (12). This important development has been discussed from the preclinical/clinical vantage points of various solid tumors by Vandenberk et al. (Stefaan W. van Gool lab) and lymphoma by Zappasodi et al. (Massimo Di Nicola lab). In the latter case, it is clear that the field is moving toward chimeric antigen receptor (CAR)-T cell’s application, and it will be interesting to see its combination with ICD in near future.

Nevertheless, the insurmountable complexity of cancer makes it inevitable that in certain contexts, ICD may fail. This failure may stem from various factors, e.g., tumor heterogeneity (8), MHC-level heterogeneity (12), pre-established niches enriched in immunosuppressive factors or immune-checkpoints (1), stem cell-based immune-evasion (12), low mutational load, inactivating mutations/polymorphisms in certain immune-receptors (1), general ablation of danger signaling (14), and other genetic or even epigenetic causes. Several of these pro-cancerous immune-evasive mechanisms and immunotherapeutic strategies required for overcoming them are discussed in detail by Kersten et al. (Karin E. de Visser lab). The strategies for targeting epigenetic processes to improve immunotherapy are further discussed by Wachowska et al. (Jakub Golab lab).

We believe that the valuable contributions of key researchers/clinicians toward this research topic/special edition have largely fulfilled its primary aim, i.e., to foster a critical discussion on experimental and clinical relevance of ICD. In fact, to further summarize and organize the fields of ICD and DAMPs, we have produced a multi-author consensus paper within this research topic that attempts to classify DAMPs and ICD inducers with an eye on translational potential of ICD (Garg et al.). This classification paper brings together >50 authors from the fields of ICD and DAMPs, and tries to reach a comprehensive accord on various terminologies related to DAMPs/ICD, the historical background of these concepts, ICD classification system (Type I vs. Type II inducers), and the relevant preclinical/clinical criteria crucial for the field(s) (Garg et al.). We hope that this consensus paper will be a useful literature resource for various researchers/clinicians. These contributions, while summarizing the *status quo*, have also exposed a set of major questions and challenges that still need to be addressed.

## Major Questions to Resolve

1. *Which danger signaling module is most specific to ICD?* Ecto-CRT seems to have remarkable exclusivity to ICD (10, 18–20) yet certain ICD inducers do not induce secreted-ATP (10), released-HMGB1 (19), or Type I IFN-responses (21). Alternatively, many non-ICD inducers induce secreted-ATP (22), released-HMGB1 (23), or Type I IFN-response (21). In fact, Type I IFN-responses can neutralize oncolytic viruses through antiviral signaling (24).
2. *Are ICD-associated DAMPs interchangeable?* Ecto-HSP90 was proposed to be interchangeable with ecto-CRT (25, 26), but this was recently invalidated in another set-up (21).
3. *Could ICD-associated DAMPs act as bystanders in certain contexts?* Induction of ICD-associated DAMPs may not always translate into a relevant functional outcome, e.g., Bleomycin induces all ICD-associated DAMPs yet elicits Tregs induction (27).
4. What is the full extent of “plasticity” of ICD-associated danger/immunogenic signaling?
5. *What is the exact role of cellular catabolic processes in regulating ICD?* Current results are highly variable; while macroautophagy positively regulates secreted-ATP (28), yet it can also negatively regulate ecto-CRT (29–31). Also, the exact roles of chaperone-mediated autophagy/CMA [CMA-essential gene *Lamp2a* regulates ecto-CRT (29)] or proteasome activity remains unresolved (Bortezomib induces ICD but not MG132, yet both inhibit the proteasome) (5).
6. *What are the common molecular determinants of ICD across various cell death pathways?* ICD-profile is largely associated with caspase-dependent apoptosis (18) but association with necroptosis is also emerging (10).
7. How does ICD counter-act the (innately) apoptosis-associated immunosuppressive processes?
8. *Does the role of ROS in ICD extend beyond a proximal stressor?* e.g., ROS-elicited oxidation-associated molecular patterns/OAMPs have been shown to mediate immunogenic potential (11).
9. *Why ICD fails in certain (GEMM) cancer mice models* (8) *but works in others* (9, 32)?
10. *Can epigenetic events* [*e.g., Long non-coding/micro-RNA* (33)] *regulate ICD and how?*

## Translational/Clinical Challenges

1. Can ICD’s clinical translation withstand the “adverse effects” of mice-to-human immune differences?
2. *Confirming ICD’s existence in a prospective (high-powered/supervised) clinical trial*.
3. *Can ICD withstand the (clinical-)operational/regulatory (GLP/GMP/GCP) hurdles associated with anticancer vaccines-production?* [indications for which are emerging (12)]
4. *Characterizing ICD-resistance mechanisms in the clinic*.
5. *Characterizing reliable ICD-biomarker(s) detectable in patient tumor/sera-samples*.
6. *Investigating ICD as a source of robust prognostic/predictive/mechanistic biomarkers* [a point investigated recently in some studies (13, 34)].

We believe that the operational function of ICD (i.e., a dying cancer cell eliciting heightened immunogenicity-driven antitumor immunity) is incontrovertibly valid; but, owing to the incomprehensible complexity of cancer, the “specifics of ICD” (i.e., its molecular, signaling, and immunological determinants) will always remain open to amenability and variations. We envisage that overtime various “variants” of ICD may emerge that differ from each other in a manner dependent upon, the type of anticancer therapy, cancer cell death pathways, cancer-types, tumor antigen make-up, the *in vivo*/*in situ* location, and the location-dependent immune-contexture.

## Author Contributions

ADG wrote the manuscript. PA provided senior supervision and critically revised the manuscript.

## Conflict of Interest Statement

The authors declare that the research was conducted in the absence of any commercial or financial relationships that could be construed as a potential conflict of interest.
